# Effects of Multi-Strain Probiotic Supplementation in Low–Crude Protein Diets on Growth Performance, Apparent Nutrient Digestibility, Fecal Microbial Indicators, and Nitrogen Utilization in Weaned Piglets

**DOI:** 10.3390/ani16050727

**Published:** 2026-02-26

**Authors:** Wei Han Zhao, Hao-Yu Liu, De-Min Cai, Dae-Kyung Kang, In Ho Kim

**Affiliations:** 1 Department of Animal Biotechnology, Dankook University, Cheonan 31187, Republic of Korea; zhaoweihan57@gmail.com (W.H.Z.); dkkang@dankook.ac.kr (D.-K.K.); 2 Smart Animal Bio Institute, Dankook University, Cheonan 31187, Republic of Korea; 3 College of Animal Science and Technology, Yangzhou University, Yangzhou 225009, China; 007725@yzu.edu.cn (H.-Y.L.); demincai@yzu.edu.cn (D.-M.C.)

**Keywords:** growth performance, apparent nutrient digestibility, fecal microbial indicators, nitrogen utilization, weaned piglets

## Abstract

This study evaluated the effects of supplementing a multi-strain probiotic (MSP) in low–crude protein (LP) diets on growth performance, apparent nutrient digestibility, selected fecal microbial populations, and nitrogen (N) utilization in weaned piglets. A 7-week feeding trial was conducted using 105 weaned crossbred piglets (Duroc × [Landrace × Yorkshire]) with an initial body weight of 6.55 ± 1.09 kg. Based on initial body weight and sex, piglets were randomly assigned to three dietary treatments using a randomized complete block design, with seven replicates per treatment and five pigs per pen (two gilts and three barrows). The dietary treatments included CON: normal crude protein (CP) diet; TRT1: LP diet; and TRT2: LP diet supplemented with 0.01% MSP. At week 7, the final body weight (BW) was higher in CON and TRT2 than in TRT1 (*p* < 0.05). During weeks 1–3, the feed conversion ratio (FCR) was lower in CON than in TRT1 (*p* < 0.05). In weeks 5–7, average daily gain (ADG) was higher in CON and TRT2 than in TRT1 (*p* < 0.05), while only CON showed a reduced FCR (*p* < 0.05). Over the entire trial, ADG was highest in TRT2 (*p* < 0.05), and FCR was lower in both CON and TRT2 compared with TRT1 (*p* < 0.01). At the end of the experiment, apparent nitrogen digestibility was significantly improved in CON and TRT2 relative to TRT1 (*p* < 0.05). Fecal *Lactobacillus* counts were elevated, and *Escherichia coli* abundance decreased in TRT2 (*p* < 0.05), indicating a more favorable profile of selected fecal bacterial indicators. In the N balance trial, six healthy piglets (three barrows and three gilts) per treatment were randomly selected and housed individually in metabolism cages. After a 3-day adaptation, total feces and urine were collected for four days to determine N intake, fecal and urinary N excretion, and N retention. At week 7, DM and N intake were unaffected by dietary treatment, whereas fecal N excretion was lower in TRT2 and CON relative to TRT1 (*p* < 0.05). Overall, supplementation of MSP in LP diets partially mitigated the adverse effects associated with reduced dietary protein by enhancing feed efficiency and nitrogen utilization, resulting in growth performance comparable to that of piglets fed a normal CP diet.

## Simple Summary

Lowering protein levels in piglet diets can reduce nitrogen pollution but may impair growth. This study shows that adding a mixture of beneficial bacteria to low-protein diets improves growth rate and feed efficiency in weaned piglets, achieving performance similar to that of pigs fed high-protein diets. Probiotic supplementation also enhanced nitrogen use, reduced nitrogen losses in manure, and modulated selected fecal bacterial indicators by increasing *Lactobacillus* counts and reducing *Escherichia coli* counts. Overall, adding probiotics to low-protein diets may represent a promising and environmentally sustainable nutritional strategy that supports piglet growth while lowering environmental nitrogen emissions.

## 1. Introduction

CP is a key nutritional component of swine diets, providing essential amino acids required for growth, tissue deposition, and metabolic functions [1]. Conventional nursery diets typically contain relatively high CP levels to maximize growth performance in weaned piglets. However, excessive dietary protein increases N excretion, exacerbates environmental pollution, and, by increasing the supply of undigested protein to the hindgut, promotes fermentation by potentially pathogenic microorganisms, thereby aggravating post-weaning digestive disturbances [2]. Therefore, reducing dietary CP while maintaining growth performance has become an important objective in modern swine nutrition.

When adequately supplemented with essential amino acids, LP diets have been shown to reduce N excretion and improve environmental sustainability [3]. Nevertheless, insufficient protein intake can limit the availability of substrates required for protein deposition and, if improperly managed, may impair apparent nutrient digestibility, intestinal function, and the stability of selected intestinal bacterial populations [4]. Consequently, the development of nutritional strategies that enhance nutrient utilization efficiency and gut health is essential to maintain piglet performance under LP feeding regimes.

Dietary supplementation with exogenous MSP has been proposed as a potential nutritional strategy to improve nutrient utilization efficiency and mitigate the adverse effects associated with LP diets [5]. MSP, which are primarily composed of *Bacillus* spp., exhibit strong environmental tolerance and a high potential for gastrointestinal colonization, allowing them to exert sustained biological functions within the digestive tract [6]. *Bacillus* spp. can secrete a wide range of digestive enzymes, including proteases and carbohydrases, thereby facilitating the breakdown of complex feed components, reducing the impact of anti-nutritional factors, and enhancing the release and utilization of nutrients in the gastrointestinal tract [7]. By promoting the degradation of dietary protein and non-starch polysaccharides, MSP supplementation may increase amino acid bioavailability, reduce endogenous nutrient losses, and decrease the flow of undigested substrates into the hindgut [8].

Beyond improvements in nutrient digestion, previous studies have demonstrated that supplementation with Bacillus-based probiotic complexes can influence selected intestinal bacterial populations through multiple mechanisms, including enhanced nutrient digestion in the upper gastrointestinal tract and competitive exclusion of harmful microorganisms [9]. Moreover, such supplementation can reduce abnormal protein fermentation in the hindgut, thereby creating a more favorable intestinal environment for the proliferation of beneficial bacteria such as *Lactobacillus* [10]. A stable and beneficial intestinal microbial environment is closely associated with intestinal integrity, nutrient utilization efficiency, and overall growth performance in weaned piglets.

N utilization efficiency represents another critical consideration in LP feeding strategies. Maximizing N digestibility and retention while minimizing fecal and urinary N excretion is of great importance for improving production efficiency and achieving environmental sustainability [11]. In addition, alterations in dietary protein level and its digestive utilization may influence systemic metabolic and endocrine responses, including indicators related to protein metabolism [12].

Therefore, the present study aimed to evaluate the effects of dietary MSP supplementation in an LP diet on growth performance, apparent nutrient digestibility, selected fecal microbial indicators (*Lactobacillus* spp. and *Escherichia coli* counts), and N retention in weaned piglets. A normal CP diet was included as a reference to determine whether MSP supplementation could compensate for the reduction in dietary protein and maintain productive performance. We hypothesized that supplementation of 0.01% MSP in an LP diet would improve feed efficiency, enhance N utilization, and favorably modulate selected fecal microbial indicators, thereby supporting growth performance that is comparable to that of piglets fed a normal CP diet.

## 2. Materials and Methods

### 2.1. Ethical Statement

The authors confirm that the ethical policies of the journal, as noted on the journal’s author guidelines page, have been adhered to and that the appropriate ethical review committee approval has been obtained. All animal experiments complied with established ethical guidelines and were approved by the Institutional Animal Care and Use Committee of Dankook University, Republic of Korea (Approval No. DK-2-2507; 14 March 2025).

### 2.2. Experimental Material

ENTEROSURE™ (Kemin Industries, Inc., Des Moines, IA, USA) is a proprietary multi-strain probiotic formulation designed to enhance gastrointestinal resilience in production animals. The product contains multiple synergistic *Bacillus* strains, including *Bacillus subtilis* PB6, *Bacillus subtilis* FxA, and *Bacillus licheniformis* G3, which contribute to the inhibition of enteric pathogens and the maintenance of stable intestinal bacterial populations. The inclusion level of 0.01% MSP in the diets was selected based on the manufacturer’s recommended dosage, and preliminary trials confirmed its feasibility and safety for weaned piglets. After feed manufacturing and mixing, the viable counts of probiotic microorganisms in the experimental diets were determined using the standard plate counting method. The results showed that the final feeds contained more than 2 × 10^8^ CFU/kg of *Bacillus subtilis* and more than 1 × 10^8^ CFU/kg of *Bacillus licheniformis*, reflecting the actual survival of the probiotics during feed processing and mixing.

### 2.3. Experimental Design, Animals, and Housing

A total of 105 weaned crossbred piglets [Duroc × (Landrace × Yorkshire)] were used in a 7-week trial. Piglets were weaned at 21 ± 1 days of age and had an initial body weight of 6.55 ± 1.09 kg at the start of the experiment. A randomized complete block design was used, with sex included as the blocking factor. Piglets were allocated to three dietary treatments, with seven replicate pens per treatment and five piglets (two gilts and three barrows) per pen.

The dietary treatments were as follows: (1) a normal CP diet (CON); (2) an LP diet (TRT1); and (3) an LP diet supplemented with 0.01% MSP (TRT2). All experimental diets were formulated to meet or exceed the nutrient requirements of weaned piglets as recommended by the National Research Council [13]. The ingredient composition and analyzed nutrient contents of the experimental diets during the three feeding phases are presented in Table 1. All piglets were housed in an environmentally controlled nursery facility with plastic-slated floors and a mechanical ventilation system. Each pen measured 1.3 × 1.3 m and was equipped with a stainless-steel automatic feeder and nipple drinkers, allowing ad libitum access to feed and water. Artificial lighting was provided for 12 h per day. The ambient temperature was maintained at approximately 30 °C during the initial phase and was gradually reduced to 24 °C as the piglets grew. Throughout the experiment, the health status of the piglets, as well as their feed and water intake, were monitored daily. Routine inspections were conducted at 08:00, 12:00, 16:00, and 20:00.

### 2.4. Growth Performance

During the 7-week trial period, the initial BW of each pig was recorded at the start of the experiment. Final body weights were recorded at weeks 1, 3, 5, and 7, based on which ADG was calculated on a pen basis. Daily feed intake and leftover feed were monitored to determine the average daily feed intake (ADFI), which was then used to calculate the FCR.

$$ADG\left(g/day\right)=\frac{Final\;BW-Initial\;BW\left(g\right)}{Experimental\;days}$$

$$ADFI\left(g/day\right)=\frac{Total\;Feed\;Intake\left(g\right)}{Experimental\;days}$$

$$FCR=\frac{Feed\;Intake\left(kg\right)}{Weight\;Gain\left(kg\right)}$$

### 2.5. Apparent Nutrient Digestibility

To determine apparent total tract digestibility (ATTD), chromium oxide (Cr_2_O_3_) was incorporated into the diets at 0.50% during the first 7 d of weeks 1, 3, 5, and 7 of the experiment as an indigestible marker. Feed samples were collected in sterile containers for subsequent analysis. Fecal samples were obtained by rectal stimulation at a consistent time each morning for three consecutive days. At each sampling time point, healthy piglets with a uniform body weight were randomly selected from each pen (one gilt and one barrow). Samples were pooled by pen prior to analysis, and data at each time point were analyzed separately. All feed and fecal samples were preserved at −20 °C until the determination of DM, N, gross energy (E), and chromium concentrations. Before laboratory analyses, all samples were oven-dried at 60 °C for 72 h, finely ground, and passed through a 1 mm sieve to ensure uniformity. Determination of apparent nutrient digestibility and chromium recovery followed previously published methodologies [14,15]. The chromium concentration was quantified using a UV–visible spectrophotometer (Optizen POP, Seoul, Republic of Korea), gross energy was measured with an adiabatic bomb calorimeter (Parr 6100, Moline, IL, USA), and *N* content was analyzed using a Kjeldahl system (Kjeltec 2300, Foss Tecator AB, Hillerød, Denmark). Apparent total tract digestibility was calculated using the following equation:

$$ATTD(\%)=[1-\{(N_{f}\times C_{d})/(N_{d}\times C_{f})\}]\times 100$$

where *N_f_* represents the nutrient concentration in feces (% DM), *C_d_* denotes the chromium concentration in the diet (% DM), *N_d_* represents the nutrient concentration in the diet (% DM), and *C_f_* denotes the chromium concentration in feces (% DM).

### 2.6. Retention and Excretion

Nitrogen balance trials were conducted during weeks 1, 3, 5, and 7 of the experiment using the total collection method. The piglets used for nitrogen balance assessment were selected from the same population as the main growth performance experiment to ensure consistency in dietary treatments, nutritional background, and management conditions.

At each designated sampling time point (weeks 1, 3, 5, and 7), six healthy weaned piglets per dietary treatment (three barrows and three gilts) were randomly selected from the respective treatment groups. The selected animals had body weights within ±5% of the treatment’s mean and showed no clinical signs of disease. Different animals were selected at each sampling time point; therefore, the measurements obtained during each sampling period were considered independent.

At each sampling time point, the selected piglets were temporarily transferred from their original pens to stainless-steel metabolism cages specifically designed to allow the separate and quantitative collection of feces and urine. The cage structure prevented cross-contamination between excreta and ensured accurate measurement of nutrient excretion.

Prior to sample collection at each time point, pigs were allowed a 3-day adaptation period in the metabolism cages while continuing to receive their respective experimental diets to minimize stress and stabilize feed intake. This was followed by a 4-day total feces and urine collection period. Upon completion of each collection period, piglets were returned to their original pens and continued in the main growth performance experiment under their assigned dietary treatments.

During the collection period, daily feed intake was recorded. Feed samples were dried at 105 °C to a constant weight for the determination of DM content. DM intake was calculated based on the daily feed intake and the DM content of the diet. N intake was calculated from the DM intake and the N concentration of the corresponding diet.

Feces were quantitatively collected and weighed daily and then stored at −20 °C. At the end of the collection period, fecal samples from each pig were pooled, dried at 60 °C for 72 h, ground, and passed through a 1 mm sieve for N analysis. Urine was collected daily into pre-acidified containers with 50 mL of 6 mol/L H_2_SO_4_ to prevent N volatilization. Total urine volume was recorded daily, and 10% aliquots were sampled proportionally and stored at −20 °C for subsequent analysis.

N concentrations in feed, feces, and urine were determined using the Kjeldahl method [15]. Fecal and urinary N excretion (g/d) were calculated based on the total excretion during the collection period. N retention (g/d) was calculated as the N intake minus the sum of fecal and urinary N excretion. The N retention rate (%) was calculated as N retention divided by the N intake and multiplied by 100.

### 2.7. Fecal Microbial Analysis

Fecal samples were pooled by pen, transported to the laboratory, and analyzed immediately. A 1 g composite sample was diluted with 9 mL of peptone broth (10 g/L) and thoroughly homogenized. Serial tenfold dilutions were prepared using 1% peptone solution and subsequently plated onto MacConkey agar (Difco Laboratories, Detroit, MI, USA) and *Lactobacilli* medium III agar (Medium 638; DSMZ, Braunschweig, Germany) for the enumeration of *Escherichia coli* and *Lactobacillus*, respectively. For each medium, three dilution levels were inoculated at a volume of 20 μL per plate, with duplicate plating. *Lactobacilli* medium III agar plates were incubated under anaerobic conditions at 39 °C for 48 h, whereas MacConkey agar plates were incubated aerobically at 37 °C for 24 h. Following incubation, colonies of *Escherichia coli* and *Lactobacillus*. were counted immediately, and bacterial populations were expressed as log_10_ colony-forming units per gram of feces (log_10_ CFU/g).

### 2.8. Statistical Analysis

Data were analyzed using one-way analysis of variance (ANOVA) in SAS 9.4 (SAS Institute Inc., Cary, NC, USA) to compare the differences among dietary treatments. Prior to statistical analysis, all data were assessed for normality using the Shapiro–Wilk test and for homogeneity of variances using Levene’s test. Microbial count data were log_10_-transformed before analysis to improve normality and stabilize variance, and statistical analyses were conducted using the transformed values. For growth performance and apparent nutrient digestibility, the pen was considered the experimental unit, and pen means were used for statistical analysis. For nitrogen balance and microbial analyses, individual animals were treated as the experimental unit. For variables measured at multiple sampling time points (weeks 1, 3, 5, and 7), data obtained at each time point were analyzed separately using one-way ANOVA. When a significant effect was detected by ANOVA, means were separated using Tukey’s multiple comparison test. Differences were considered statistically significant at *p* < 0.05. All animals that completed the entire experimental period were included in the statistical analysis, and no data were excluded.

## 3. Results

The effects of MSP supplementation in LP diets on the growth performance of weaned piglets are presented in Table 2. No significant differences were observed among treatments in initial body weight or body weight during the early stages of the experiment (*p* > 0.05). However, at week 7, the final body weight was significantly greater in the CON and TRT2 groups than in the TRT1 group (*p* < 0.05).

During weeks 1–3, the FCR in the CON group was significantly lower than that in the TRT1 group (*p* < 0.05), whereas no significant difference was observed between the TRT2 and TRT1 groups (*p* > 0.05). During weeks 5–7, ADG was significantly higher in both the CON and TRT2 groups compared with the TRT1 group (*p* < 0.05), while a significant reduction in FCR was observed only in the CON group (*p* < 0.05).

Over the entire experimental period, ADG was significantly greater in the TRT2 group than in the TRT1 group (*p* < 0.05). In addition, FCR was significantly lower in both the CON and TRT2 groups relative to the TRT1 group (*p* < 0.05), indicating that MSP supplementation partially improved growth efficiency under LP dietary conditions.

As shown in Table 3, during weeks 1, 3, and 5, no significant differences were observed among treatments in DM, N, or E digestibility (*p* > 0.05). At the end of the experiment, N digestibility was significantly higher in the CON and TRT2 pigs compared with the TRT1 pigs (*p* < 0.05), while DM and E digestibility remained unaffected (*p* > 0.05).

As shown in Table 4, dietary treatments had no significant effects on fecal microbial populations during weeks 1, 3, and 5 (*p* > 0.05). At week 7, pigs in the TRT2 group exhibited a significantly higher fecal Lactobacillus count and a significantly lower *Escherichia coli* count compared with the TRT1 group (*p* < 0.05), indicating that MSP supplementation contributed to an improvement in gut microbial balance.

As shown in Table 5, no significant differences were observed among treatments during weeks 1, 3, and 5 (*p* > 0.05). At week 7, fecal N excretion was significantly lower in CON and TRT2 compared with TRT1 (*p* < 0.05). This indicated that MSP supplementation in an LP diet improved nitrogen utilization during the later stage of the experiment.

## 4. Discussion

Growth performance is critical for the post-weaning growth and development of weaned piglets, as it integrally reflects dietary nutrient intake, digestive and absorptive capacity, and metabolic utilization [16]. Previous studies have demonstrated that probiotic feed additives can improve nutrient utilization efficiency without significantly increasing ADFI, thereby compensating for the adverse effects associated with reduced dietary protein levels [17]. In particular, multi-strain Bacillus-based additives have been shown to significantly increase ADG and reduce FCR in nursery pigs [18]. The present findings are generally consistent with these reports. Supplementation of MSP in LP diets increased ADG and reduced FCR during the late growth phase, resulting in an overall growth performance comparable to that of piglets fed a normal CP diet. Moreover, no significant differences in ADFI were observed among treatments, suggesting that the improvement in growth performance was more likely attributable to enhanced feed efficiency rather than increased feed intake per se. Under LP conditions, growth efficiency improvements are often linked to enhanced digestive function, reduced amino acid deamination, and better intestinal health [19]. They may have partially contributed to more efficient nutrient conversion into body tissue.

Apparent nutrient digestibility, particularly N utilization efficiency, is an important determinant of protein utilization in weaned piglets [20]. Previous studies have reported that Bacillus-based probiotics can enhance ileal amino acid digestibility in weaned pigs, and improvements in N digestibility contribute to a reduction in the flow of undigested protein into the hindgut, thereby decreasing proteolytic fermentation and N losses [7]. In the present study, no significant differences were observed among treatments in dry matter DM and E digestibility during the early and middle phases of the experiment, suggesting that the reduction in dietary protein level did not adversely affect overall apparent nutrient digestibility under the current experimental conditions. However, at the end of the trial, MSP supplementation in the LP diet significantly improved N digestibility, reaching a level comparable to that of the normal CP group. This improvement was more evident during the later growth phase. As digestive enzyme secretion and intestinal absorptive capacity progressively mature with age, the potential modulatory effects of feed additives may become more apparent; nevertheless [21].

The intestinal bacterial populations represent a critical biological link connecting dietary modulation, nutrient utilization, and host growth performance [22]. Previous studies have demonstrated that *Bacillus* supplementation can increase beneficial bacterial populations while suppressing the proliferation of potential pathogens in weaned pigs [23]. Consistent with these findings, the present study showed that MSP supplementation at the end of the experimental period significantly increased fecal *Lactobacillus* abundance while reducing *Escherichia coli* counts. *Lactobacillus* spp. can enhance intestinal barrier function, competitively inhibit pathogen adhesion, and produce organic acids that lower intestinal pH, thereby creating a more favorable intestinal environment for nutrient digestion and absorption [24]. In contrast, reductions in *Escherichia coli* abundance may alleviate subclinical inflammatory responses and decrease endogenous nutrient losses [25]. It is noteworthy that significant changes in microbial populations were observed only at week 7 in the present study. This may suggest that the modulatory effects of MSP require sustained supplementation to become apparent. In addition, variations in dietary composition across different phases may influence substrate availability and the intestinal microbial metabolic environment. The structure and function of the gastrointestinal tract in weaned piglets gradually mature during development; changes in barrier function, digestive enzyme secretion, and microbial stability may all affect probiotic colonization and functional outcomes [26]. Furthermore, limitations related to sample size and statistical power may have reduced the ability to detect subtle differences during earlier stages. Therefore, the absence of significant effects in the early phase does not necessarily exclude the possibility of underlying biological influences. The microbial analysis in this study primarily relied on culture-based enumeration of Lactobacillus spp. and Escherichia coli, which cannot fully capture the complexity of the intestinal microbiota. Future studies using high-throughput sequencing techniques, such as 16S rRNA sequencing, would help to further explore the broader effects of MSP on gut microbial communities.

N retention and excretion parameters are key indicators for evaluating protein utilization efficiency and the environmental sustainability of swine production [27]. It has been reported that dietary probiotic supplementation can reduce N excretion and enhance N retention efficiency in growing pigs [28]. In addition, supplementation with multi-strain Bacillus has been shown to improve gut health and growth efficiency in weaned pigs [29]. In the present study, no significant differences in N balance parameters were observed during the early and middle post-weaning phases. However, at week 7, MSP supplementation significantly reduced fecal N excretion and was associated with numerically lower urinary N excretion and higher N retention. The reduction in fecal N excretion directly reflects improved intestinal N digestibility, which is consistent with the digestibility results observed in this study. Moreover, the tendency toward reduced urinary N excretion may indicate a moderation of excessive amino acid deamination and urea synthesis [30]. These processes are typically elevated when protein supply exceeds the animal’s metabolic requirements [31]. Collectively, these findings suggest that MSP supplementation may improve nitrogen utilization efficiency, potentially through effects on intestinal digestive function, microbial modulation, and systemic nitrogen metabolism. It should be emphasized that this study was conducted under a feeding program in which dietary crude protein levels were progressively reduced across different phases. Therefore, the improvements observed during the late nursery period may reflect the combined influences of probiotic supplementation, physiological maturation of piglets, and dietary adjustments implemented during different feeding phases. Accordingly, the conclusions of the present study are more applicable to LP feeding systems based on multiple feeding phases and should not be directly extrapolated to a single diet with a fixed protein level. Future studies employing mixed model or repeated measures statistical approaches are warranted to further clarify the interactions among these factors.

## 5. Conclusions

In summary, supplementation of 0.01% MSP in a LP diet partially alleviated the potential adverse effects associated with reduced dietary protein levels. MSP supplementation was associated with improvements in feed efficiency, enhanced nitrogen digestibility, and favorable shifts in selected fecal bacterial populations, while growth performance did not differ significantly from that of piglets fed a normal crude protein diet. These results indicate that MSP may serve as a promising nutritional strategy to improve nitrogen utilization efficiency while maintaining growth performance, thereby potentially contributing to reduced nitrogen excretion and improved environmental sustainability in swine production.

## Figures and Tables

**Table 1:** Composition of weaning pig diets.

Item	Phase 1 (Week 1–2)		Phase 2 (Week 3–4)		Phase 3 (Week 5–7)	
	PC (Basal Diet)	NC (CP −2%)	PC (Basal Diet)	NC (CP −2%)	PC (Basal Diet)	NC (CP −2%)
Ingredients (%)						
Corn	41.59	46.55	56.45	61.38	65.93	70.91
Soybean meal	18.70	13.65	15.19	10.16	18.40	13.35
Fermented soybean meal	10.00	10.00	8.00	8.00	5.00	5.00
Whey protein	10.00	10.00	6.00	6.00	2.00	2.00
Soy oil	1.68	1.66	1.79	1.77	0.98	0.94
Lactose	13.38	13.38	8.03	8.03	3.73	3.73
Monocalcium phosphate	1.45	1.57	1.35	1.47	1.14	1.26
Limestone	0.89	0.88	0.91	0.91	0.83	0.82
Lysine (78%)	0.81	0.81	0.81	0.81	0.66	0.66
Methionine (98%)	0.20	0.20	0.17	0.17	0.12	0.12
Threonine (98%)	0.31	0.31	0.30	0.30	0.23	0.23
Tryptophan (98%)	0.04	0.04	0.05	0.05	0.03	0.03
Salt	0.20	0.20	0.20	0.20	0.20	0.20
Mineral mix ^1^	0.20	0.20	0.20	0.20	0.20	0.20
Vitamin mix ^2^	0.20	0.20	0.20	0.20	0.20	0.20
ZnO	0.25	0.25	0.25	0.25	0.25	0.25
Choline (25%)	0.10	0.10	0.10	0.10	0.10	0.10
Total	100.00	100.00	100.00	100.00	100.00	100.00
Calculated value						
Crude protein, %	20.00	18.00	18.00	16.00	18.00	16.00
Metabolizable Energy, kcal/kg	3400	3400	3400	3400	3350	3350
Calcium, %	0.85	0.85	0.80	0.80	0.70	0.70
Phosphorus, %	0.70	0.70	0.65	0.65	0.60	0.60
Lysine, %	1.70	1.56	1.53	1.40	1.40	1.27
Methionine, %	0.49	0.46	0.44	0.42	0.40	0.38
Threonine, %	1.05	0.97	0.95	0.87	0.87	0.79
Tryptophan, %	0.28	0.25	0.25	0.22	0.23	0.20
Crude Fat, %	3.72	3.79	4.21	4.29	3.68	3.74
Crude Fiber, %	1.90	1.81	1.99	1.89	2.20	2.10
Crude Ash, %	5.63	5.46	5.07	4.92	4.59	4.43
Lactose, %	20.00	20.00	12.00	12.00	5.00	5.00

^1^ Provided per kg diet: Fe, 100 mg as ferrous sulfate; Cu, 17 mg as copper sulfate; Mn, 17 mg as manganese oxide; I, 0.5 mg as potassium iodide; and Se, 0.3 mg as sodium selenite; ^2^ Provided per kilograms of diet: vitamin A, 10,800 IU; vitamin D_3_, 4000 IU; vitamin E, 40 IU; vitamin K_3_, 4 mg; vitamin B_1_, 6 mg; vitamin B_2_, 12 mg; vitamin B_6_, 6 mg; vitamin B_12_, 0.05 mg; biotin, 0.2 mg; folic acid, 2 mg; niacin, 50 mg; D-calcium pantothenate, 25 mg.

**Table 2:** Effect of MSP supplementation in LP diets on growth performance in weaned piglets. ^1^

Items	CON	TRT1	TRT2	SEM ^2^	*p*-Value
Body weight, kg					
Initial	6.55	6.56	6.55	0.003	0.584
Week 1	8.34	8.24	8.30	0.04	0.361
Week 3	13.91	13.43	13.75	0.15	0.109
Week 5	21.38	20.34	21.19	0.33	0.125
Week 7	31.14 ^a^	28.74 ^b^	30.98 ^a^	0.67	0.045
Initial–Week 1					
ADG, g	256	241	249	6	0.353
ADFI, g	274	287	278	7	0.394
FCR	1.073	1.198	1.116	0.005	0.152
Week 1–Week 3					
ADG, g	397	371	389	9	0.132
ADFI, g	506	486	499	13	0.621
FCR	1.273 ^b^	1.309 ^a^	1.281 ^ab^	0.011	0.039
Week 3–Week 5					
ADG, g	533	492	530	17	0.291
ADFI, g	798	758	796	22	0.438
FCR	1.497	1.549	1.503	0.020	0.195
Week 5–Week 7					
ADG, g	697 ^a^	600 ^b^	699 ^a^	26	0.039
ADFI, g	1193	1095	1215	38	0.104
FCR	1.717 ^b^	1.829 ^a^	1.747 ^ab^	0.035	0.048
Overall					
ADG, g	502 ^ab^	452 ^b^	498 ^a^	14	0.044
ADFI, g	754	707	757	18	0.174
FCR	1.504 ^b^	1.565 ^a^	1.521 ^b^	0.013	0.009

^1^ Abbreviation: MSP, multi-strain probiotic; CON, normal crude protein diet; TRT1, low crude protein diet; TRT2, TRT1 + MSP 0.01%; LP, low protein; ADG, average daily gain; ADFI, average daily feed intake; FCR, feed conversion ratio. ^2^ Standard error of means. ^a,b^ Means in the same row with different superscripts differ (*p* < 0.05).

**Table 3:** Effect of MSP supplementation in LP diets on apparent nutrient digestibility in weaned piglets. ^1^

Items, %	CON	TRT1	TRT2	SEM ^2^	*p*-Value
Week 1					
Dry matter	87.76	86.31	87.52	0.60	0.312
Nitrogen	82.66	81.93	82.34	0.63	0.762
Energy	85.69	83.91	85.17	0.69	0.333
Week 3					
Dry matter	84.85	84.55	84.78	0.60	0.931
Nitrogen	80.92	79.99	80.55	0.71	0.685
Energy	83.12	82.28	82.62	0.60	0.214
Week 5					
Dry matter	83.73	82.60	83.37	0.67	0.143
Nitrogen	78.49	76.90	77.82	0.71	0.436
Energy	80.05	79.68	81.37	0.70	0.091
Finish					
Dry matter	80.23	79.54	81.27	0.71	0.214
Nitrogen	75.83 ^a^	74.19 ^b^	75.68 ^a^	0.95	0.019
Energy	78.53	78.18	79.40	0.82	0.553

^1^ Abbreviation: MSP, multi-strain probiotic; CON, normal crude protein diet; TRT1, low crude protein diet; TRT2, TRT1 + MSP 0.01%; LP, low protein. ^2^ Standard error of means. ^a,b^ Means in the same row with different superscripts differ (*p* < 0.05).

**Table 4:** Effect of MSP supplementation in LP diets on fecal microbial in weaned piglets. ^1^

Items, log_10_ CFU/g	CON	TRT1	TRT2	SEM ^2^	*p*-Value
Week 1					
*Lactobacillus*	6.27	6.25	6.33	0.11	0.659
*Escherichia coli*	5.57	5.62	5.55	0.14	0.918
Week 3					
*Lactobacillus*	7.51	7.36	7.53	0.07	0.119
*Escherichia coli*	6.69	6.86	6.71	0.08	0.301
Week 5					
*Lactobacillus*	8.12	8.02	8.19	0.16	0.236
*Escherichia coli*	6.92	6.95	6.81	0.13	0.414
Finish					
*Lactobacillus*	8.72 ^ab^	8.67 ^b^	8.82 ^a^	0.14	0.034
*Escherichia coli*	7.46 ^ab^	7.51 ^a^	7.37 ^b^	0.12	0.046

^1^ Abbreviation: MSP, multi-strain probiotic; CON, normal crude protein diet; TRT1, low crude protein diet; TRT2, TRT1 + MSP 0.01%; LP, low protein. ^2^ Standard error of means. ^a,b^ Means in the same row with different superscripts differ (*p* < 0.05).

**Table 5:** Effect of MSP supplementation in LP diets on retention and excretion in weaned piglets. ^1^

Items, %	CON	TRT1	TRT2	SEM ^2^	*p*-Value
Week 1					
DM intake, g/d	274.7	269.8	273.2	1.37	0.291
N intake, g/d	5.57	5.65	5.67	0.02	0.184
Fecal N excretion, g/d	0.47	0.42	0.35	0.03	0.316
Urine N excretion, g/d	2.38	2.33	2.3	0.02	0.381
N retention, g/d	2.72	2.90	3.02	0.05	0.125
N retention rate, %	48.76	51.31	53.22	0.83	0.139
Week 3					
DM intake, g/d	490.2	490.7	479.2	3.14	0.084
N intake, g/d	10.6	10.5	10.1	0.08	0.061
Fecal N excretion, g/d	1.3	1.2	1.1	0.06	0.754
Urine N excretion, g/d	3.3	3.2	3	0.08	0.299
N retention, g/d	6	6.1	5.9	0.11	0.812
N retention rate, %	56.5	57.83	58.53	0.94	0.735
Week 5					
DM intake, g/d	804	811.5	801.7	6.29	0.772
N intake, g/d	16.8	16.5	16.6	0.16	0.809
Fecal N excretion, g/d	2.5	2.4	2.3	0.06	0.481
Urine N excretion, g/d	3.5	3.4	3	0.14	0.298
N retention, g/d	10.8	10.7	11.3	0.25	0.653
N retention rate, %	64.3	64.81	68.02	1.02	0.317
Week 7					
DM intake, g/d	1185	1120	1200	19.2	0.118
N intake, g/d	23.5	22.2	23.8	0.32	0.104
Fecal N excretion, g/d	2.9 ^b^	3.3 ^a^	2.8 ^b^	0.08	0.021
Urine N excretion, g/d	4.8	4.9	4.5	0.17	0.094
N retention, g/d	15.8	14	16.5	0.38	0.071
N retention rate, %	67.2	63.1	69.3	1.25	0.068

^1^ Abbreviation: MSP, multi-strain probiotic; CON, normal crude protein diet; TRT1, low crude protein diet; TRT2, TRT1 + MSP 0.01%; LP, low protein; DM, dry matter; N, nitrogen. ^2^ Standard error of means. ^a,b^ Means in the same row with different superscripts differ (*p* < 0.05).

## Data Availability

The data presented in this study are available on request from the corresponding author.

## Abbreviations

The following abbreviations are used in this manuscript:

MSP	Multi-Strain Probiotics
LP	Low Protein
N	Nitrogen
CP	Crude Protein
DM	Dry Matter
E	Energy
FCR	Feed Conversion Ratio
ADG	Average Daily Gain
ADFI	Average Daily Feed Intake
AOAC	Association Of Official Analytical Chemists
ATTD	Apparent Total Tract Digestibility
BW	Body Weight
